# Serum concentration of vascular endothelial growth factor and depth of trophoblastic invasion in ampullary ectopic pregnancy

**DOI:** 10.6061/clinics/2016(12)04

**Published:** 2016-12

**Authors:** Fábio Roberto Cabar, Décio Roberto Kamio Teshima, Pedro Paulo Pereira, Leila Antonangelo, Regina Schultz, Rossana Pulcineli Francisco

**Affiliations:** I Departamento de Obstetr�cia e Ginecologia, Faculdade de Medicina da Universidade de São Paulo, São Paulo/SP, Brazil; II Departamento de Patologia e Pesquisa Médica, Laboratório 03, Faculdade de Medicina da Universidade de São Paulo, São Paulo/SP, Brazil; III Departamento de Patologia, Faculdade de Medicina da Universidade de São Paulo, São Paulo/SP, Brazil

**Keywords:** Pregnancy First Trimester, Ectopic Pregnancy, Vascular Endothelial Growth Factor A

## Abstract

**OBJECTIVE:**

To evaluate the association between the depth of trophoblastic infiltration and serum vascular endothelial growth factorconcentration in patients with an ampullary pregnancy.

**METHODS:**

This prospective cross-sectionalstudy involved 34 patients with an ampullary ectopic pregnancy who underwent salpingectomy between 2012 and 2013. Maternal serum vascular endothelial growth factor concentrations were measured using Luminex technology. Trophoblastic invasion was classified histologically as follows: stage I, limited to the tubal mucosa; stage II, reaching the muscle layer; and stage III,involving the full thickness. The qualitative data were compared using Fisher's exact test. The nonparametric Kruskal-Wallis and Mann-Whitney tests were used to evaluate differences in serum vascular endothelial growth factor among the degrees of trophoblastic invasion. ROC curves were constructed to determine vascular endothelial growth factor cut-off values that predict the degree of tubal invasion based on the best sensitivity and specificity.

**RESULTS:**

Eight patients had stage I trophoblastic invasion, seven had stage II, and 19 had stage III. The median serum vascular endothelial growth factorconcentration was 69.88 pg/mL for stage I, 14.53 pg/mL for stage II and 9.08 pg/mL for stage III, with a significant difference between stages I and III. Based on the ROC curve, a serum vascular endothelial growth factor concentration of 25.9 pg/mL best differentiated stage I from stages II and III with asensitivity of 75.0%, specificity of 76.9%, and area under the curve of 0.798.

**CONCLUSIONS:**

The depth of trophoblastic penetration into the tubal wall isassociated with serum vascular endothelial growth factor concentration in ampullary pregnancies.

## INTRODUCTION

Ectopic pregnancy (EP) is a form of anomalous pregnancy in which the fertilized egg implants outside the uterine cavity 1. The Fallopian tube is the most common site of implantation (98%), but EP can also occur at other sites, such as the ovaries, abdomen and broad ligament 2. EPs account for 1-2% of all pregnancies, and hemorrhage due to tubal rupture remains the most common cause of maternal mortality in the first trimester of gestation 3. The increasing incidence of EP is strongly associated with a greater prevalence of late primiparity, pelvic inflammatory disease and other sexually transmitted diseases, tubal sterilization and subsequent attempted reversal, and cesarean section delivery; a larger number of pregnancies conceived using assisted reproductive technology; and the use of levonorgestrel as an emergency contraceptive method 4-6.

Vascular endothelial growth factor (VEGF) is an angiogenic factor that participates in the processes ofvascular development in the embryo and implantation and placentation, in which it functions as animportant modulator of vascular growth, remodeling and permeability in the decidua, endometrium and trophoblast 7,8. Cellular production of VEGF isaugmentedby tissue hypoxia 9,10; the implantation conditions in the tubesare very different from those in the well-vascularized endometrium, and VEGF production and secretion appear to be increased in EP 11,12.

The implantation of trophoblastic tissue into the tubal wall can damage oviductal function by evoking an inflammatory response that injures the cilia of the epithelium and disturbs the architecture of the wall musculature, thus increasing the risk for another episode of EP 13,14. The impairment of tubal function depends on the degree of trophoblast invasion into the wall of the uterine tubes. There are no adequate standardsor methods for predicting the depth of trophoblastic invasion into the tubal wall. Serum concentrations of beta-human chorionic gonadotropin (hCG) and the presence of an embryo with a heartbeat can be used to estimate trophoblastic invasion and damage to the histological structure of the Fallopian tubes caused by trophoblastic implantation 15-18. According to Cabar et al. 19, greater VEGF concentrations allow deeper invasion of trophoblastic tissue into the tubes. These authors concluded that higher serum VEGF levels are related to the discovery of an embryo with a heartbeat by ultrasound. Higher VEGF production is likely to create developmental conditions that are more favorable for ectopic embryos 20.

The objective of this study was to evaluate the association between the depth of trophoblastic infiltration into the tubal wall and serum concentrations of VEGF in patients withan ampullary pregnancy using a more sensitive method than those used previously.

## MATERIALS AND METHODS

Patients with an ampullary pregnancy who underwent a salpingectomy between July 11, 2012, and August 19, 2013, were included in this prospective study. The other inclusion criteria were singleton pregnancy, spontaneous conception, a diagnosis of tubal pregnancy in the ampullary portion and salpingectomy as the treatment. The exclusion criteria were as follows: no consensus regarding the location of the tubal pregnancy upon surgical identification and histological analysis and inability to measure serum VEGF on the day of surgery. Gestational age was determined based on the last menstrual period.

Sixty-three consecutive cases of EP were documented during the study period. Of these, 21 patients were not included for different reasons: informed consent could not be obtained from one patient, and the other 20 patients were treated by a conservative approach (clinical or surgical). Forty-two patients who met the inclusion criteria were selected to participate in the study. Eight patients were excluded: three because the tubal implantation site could not be identified, two because no trophoblastic tissue was detected by histological analysis, one because tissue VEGF could not be identified, and two because blood collectionfor serum VEGF measurement was not possible.

Routine measurements of serum beta-hCG and transvaginal ultrasounds were performed to confirm the diagnosis. After diagnostic confirmation, if the woman was eligible for inclusion in the study, the serum VEGF concentration was determined. Blood samples were collected by peripheral venous puncture into siliconized tubes. After coagulation of the sample for 30 minutes, serum was separated from whole blood by centrifugation at 4500 rpm for 20 minutes at 4°C, divided into two aliquots, and stored in a freezer at -70°C for later analysis.

The samples were analyzed in duplicate using the Bio-Plex Pro^TM^ Human Cytokine 27-plex Assay according to the manufacturer’sinstructions. The assays were carried out by staff members who were unawareof the clinical condition of each pregnant woman. The reactions were diluted 1:2 so that the results were within the measurable range of the standard curves of the kit. TheBio-Plexassay was performed to simultaneously quantify multiple cytokines in plasma. The Luminexassay uses a similar principle to that of sandwich ELISA. Colored and fluorescent microspheres/beads were coated with monoclonal antibodies against eight targetsand added to the wells. Samples and standards (ranging from 0.1 to 10,000 pg/mL per analysis) were added to the wells and the mixtures were incubated. Then, the wells were washed and the washing solution was removed with a magnetic washer in which the microspheres were magnetically retained (Bio-Plex Pro II Wash Station). After washing, a mixture of the secondary biotinylated antibody was added and the plates were incubated again. Streptavidin conjugated to a fluorescent protein, R-phycoerythrin (streptavidin-RPE), was added and the plates were incubated for a short period. After a washing step to remove unbound reagent, buffer solution (Luminex, MiraiBio, Alameda, CA) was added to the wells, and the results were recorded (100 minutes to complete all analyses) in a microsphere analyzer (Bio-Plex 200). The concentrations of the unknown samples (antigens in plasma samples) were estimated from the standard curves using Bio-Plex Manager Software (Bio-Rad Laboratories, Hercules, CA). Cytokine levels are expressed as pg/mL.

The Fallopian tubes were fixed in 10% formalin and cut into serial sections for light microscopy analysis. An average of 10 hematoxylin-eosin-stained sections were analyzed. Additionally, the histological material was stained with Masson’s trichrome to detect muscle fibers in order to facilitate the identification of tissue invaded by the trophoblast. Immunohistochemical staining for human placental lactogen was performed to identify the intermediate trophoblast and to determine the depth of trophoblastic invasion into the tubal wall. A single experienced pathologist who was unaware of the clinical and laboratory characteristics of the patients performed all the histological assessments.

Tubal pregnancies were classified histologically according to the depth of trophoblastic infiltration into the tubal wall 17: stage I, trophoblastic infiltration limited to the tubal mucosa; stage II, trophoblastic infiltration extending to the tubal muscularis; and stage III, complete tubal wall infiltration with or without rupture of the serosa.

To calculate the sample size, the study by Cabar et al. 19 was considered as a reference for the effect size for the three degrees of trophoblastic invasion. Therefore, when considering a significance level of 5% and a statistical power of 95%, the calculated minimum sample size was 21 cases (G*Power software, version 3.1.9.2).

The qualitative data are reported asthe absolute and relative frequency (percentage), and these data were compared using Fisher's exact test. As a summary of the quantitative variables, the median, minimum, maximum, mean, and standard deviation were calculated. Nonparametric Kruskal-Wallis and Mann-Whitney tests were used to evaluate differences in serum VEGFamong the degrees of trophoblastic invasion. ROC curves were constructed to determine VEGF cut-off values that predict the degree of tubal invasion based on the best sensitivity and specificity. A *p-*value<0.05 was considered to indicate statistical significance. All tests were twotailed. The statistical analysis was performed using SPSS Statistics 22.0 for Macintosh (IBM Corp., Armonk, NY).

### Ethics

The Institutional Review Board (CAPPesq-Clinical Research Ethics Committee of the University of São Paulo) approved the study. Informed consent was obtained from each patient before inclusion.

## RESULTS

Patient age ranged from 11 to 40 years (29.8±6.6 years), andthere were no significant differences in mean maternal age among the three histological groups (*p*=0.706). Twenty-four (70.4%) patients were Caucasian, and 14 (29.4%) were non-Caucasian. With respect to obstetric history, 18 (52.9%) patients were nulliparous, and five (14.7%) had a history of EP in the contralateral Fallopian tube. Histological analysis showed that eight (23.5%) patients had stage I tubal infiltration, seven (20.6%) had stage II, and 19 (55.9%) had stage III. The gestational age ranged from 28 to 95 days (53.2±14.5 days), and there were no significant differences in the mean gestational ageamong the three histological groups (*p*=0.604).

Serum VEGF concentrations on the day of surgery ranged from 0.036 to 205.16 pg/mL (median, 14.77 pg/mL).The Kruskal-Wallis test revealed that at least one stage was significantly different from the others (*p*=0.036) (Table 1).

Pairwise comparisons of VEGF concentration among the three stages of trophoblastic invasion revealed a significant difference between stage I and stage III (*p*=0.031); stage I invasion was associated with higher median VEGF values than those in stage III (Table 1). On the other hand, no differences were found when patients with stage I *vs*. stage II and stage II *vs*. III were compared, demonstrating that serum VEGF can only differentiate patients at stage I from those at stage III (Table 2).

As there was no significant difference between stage I and stage II or between stage II and stage III, an analysis was performed after grouping stages II and III (II + III) or stages I and II (I+II). The first comparison showed a significant difference (*p*=0.010): stage I cases presented a higher median serum VEGF concentration compared to stage II + III cases. On the other hand, the second comparison showed no significant difference between stage III and stages I + II (*p*=0.05) (Table 3). Based on the previous finding of a difference only between stage I and stages II + III, the ROC curve was adjusted to obtain a cut-off concentration of VEGF that better discriminated stage I from stages II + III, with better sensitivity and specificity values. A serum VEGF level of 25.9 pg/mL best differentiated stage I from stages II +III, with a sensitivity of 75.0%, specificity of 76.9%, and area under the curveof 0.798.

## DISCUSSION

The results revealed a difference in serum VEGF concentration between patients with stage I and stage III trophoblastic invasion, with stage I cases presenting with a higher median VEGF concentration than stage III cases. No differences were found for stage I *vs*. stage II or for stage II *vs*. III; thus, serum VEGF can only differentiate stages I and III. ROC curve analysis showed that a serum VEGF level of 25.9 pg/mL best differentiated stage I from stages II + III, with a sensitivity of 75.0% and a specificity of 76.9%. A previous study from our group demonstrated an increase in the depth of trophoblastic penetration into the tubal wall with increasing maternal serum VEGF concentration 19. We postulated that elevated cellular production of VEGF permits deeper invasion of trophoblastic tissue into the tubal wall. In the present study, we intended to repeat the investigation using a more sensitive method for measuring serum VEGF, namely, Luminextechnology, which is similar in principle to sandwich ELISA.

Unexpectedly, serum VEGF concentrations decreased with the depth of trophoblastic invasion into the tubal wall; lower VEGF levels were observed in cases with deeper invasion. A previous study with a similar population size reported that a cut-off value of 297.2 pg/mL predicted stage I trophoblastic invasion with 100% sensitivity and a negative predictive value of 100%. Moreover, VEGF levels above 440.1 pg/mL predicted stage III trophoblastic invasion. Considering the high sensitivity, specificity and positive and negative predictive values, higher serum VEGF levels seemed to be a good predictor of deeper trophoblast invasion and consequently greater destruction of the tubal wall 19. Serum VEGF levels were higher in cases of more superficial trophoblastic invasion. The findings of the present study are different from those reported by Cabar et al. 19, who showed a direct relationship between increased serum VEGF and the depth of trophoblastic invasion into the oviduct. We believe that the use of different methods for determining serum VEGF levels may have contributed to the differences between the studies.

Only ampullary pregnancies were included in this study since they are the most common extrauterine site of trophoblastic implantation, accounting for more than 70% of all EPs^21^. Sincethe anatomical segments of the Fallopian tube differ histologically, trophoblastic penetration may be different in each tubal portion.

In the population studied, trophoblastic invasion was limited to the mucosa in 23.5% of the patients, reached the muscle layer in 20.6%, and penetrated the full thickness of the tubal wall in the remaining 55.9%. Similar percentages were reported by Cabar et al. 15 (27.6%, 28.6% and 43.8%, respectively). The predominance of cases in which trophoblastic invasion reached at least the muscle layer (76.5%) is consistent with previous studies 15,17,20.

Serum progesterone concentrations are increased in pregnancy and there is an important relationship between increases in hCGand VEGF that contributes to trophoblastic invasion. Evans et al. 10 stated that progesterone enhances VEGF production in epithelial cells of the retina and that hCG increases VEGF in granulosa cells. Since the serum levels of progesterone and hCG are lower in EP, despite local hypoxia, VEGF production may remain unchanged due to the negative effect of the decreases in these hormones.

Previous studies have shown that estrogen increases VEGF secretion in humans and animals 22,23. Similarly, reductions in the serum levels of estrogen, progesterone and hCG, in addition to decreases in other cytokines and growth factors with a yet unknown role in EP, may increase the concentration of VEGF, albeit not to a significant degree due to this counterbalancing effect.

In this study, we aimed to determine why trophoblastic invasion encounters more favorable conditions and reaches the deepest layers of the Fallopian tube in some cases. An understanding of this process and its relationship with possible predictors of trophoblastic invasion in the Fallopian tubes, such as serum beta-hCG and ultrasound findings,should contribute to the diagnosis and treatment of women with EP. We decided to study serum concentrations because we intended to provide information to clinicians that could help them decide whether to remove Fallopian tubes affected by EP (conservative or radical treatment).

Due to differences between the two existing studies on this subject, we believe that additional studies with a larger number of cases are necessary to completely clarify this issue.

We conclude that the depth of trophoblastic invasion into the tubal wall is associated with maternal serum VEGF concentration in women with ampullary EP. VEGF can discriminate stage I from stage III cases in terms of penetration depth.

Our results differ from those of previous studies, and further investigation is needed to confirm our findings.

## AUTHOR CONTRIBUTIONS

Cabar FR (main researcher) conceived, designed and performed the study.Teshima DR collected data and wrote the manuscript. Pereira PP analyzed the data. Antonangelo L performed the experiments. Schultz R performed the pathological analysis. Francisco RP analyzed the data and revised the manuscript.

## Figures and Tables

**Table 1:** Serum VEGF and depth of trophoblastic invasion.

Trophoblastic invasion	Number of patients	VEGF (pg/mL) Median (range)
Stage I	8	69.88 (5.30–93.46)
Stage II	7	14.53 (0.36–68.16)
Stage III	19	9.08 (0.0–205.16)

*p*=0.036 (nonparametric Kruskal-Wallis test).

**Table 2:** Pairwise comparison of the depth of trophoblastic invasion.

Comparison	p-value*
Stage I *vs.*stage II	0.310
Stage I *vs*.stage III	0.031
Stage II *vs*.stage III	0.990

* Nonparametric Kruskal-Wallis test.

**Table 3:** Serum VEGF and depth of trophoblastic invasion.

Trophoblastic invasion	Number of patients	VEGF (pg/mL) Median (range)	p-value*
Stage I *vs*.	8	69.88 (5.30–93.46)	0.010
Stage II + III	26	11.34 (0.0–205.16)	
Stage III *vs*.	19	9.08 (0.0–205.16)	0.050
Stage I + II	15	29.59 (0.36–93.46)	

* Mann-Whitney test.
